# MicroMagnify: a multiplexed expansion microscopy method for pathogens and infected tissues

**DOI:** 10.21203/rs.3.rs-2637060/v1

**Published:** 2023-03-06

**Authors:** Yongxin Zhao, Zhangyu Cheng, Caroline Stefani, Thomas Skillman, Aleksandra Klimas, Aramchan Lee, Emma DiBernardo, Karina Mueller Brown, Tatyana Milman, Brendan Gallagher, Katherine Lagree, Bhanu Jena, Jose Pulido, Scott Filler, Aaron Mitchell, Luisa Hiller, Adam Lacy-Hulbert

**Affiliations:** Carnegie Mellon University; Carnegie Mellon University; Benaroya Research Institute; Immersive Science LLC; Carnegie Mellon University; Carnegie Mellon University; Carnegie Mellon University; Carnegie Mellon University; Wills Eye Hospital and Jefferson University Hospital; Carnegie Mellon University; Carnegie Mellon University; Wayne State University School of Medicine; Wills Eye Hospital and Jefferson University Hospital; Lundquist Institute for Biomedical Innovation at Harbor-UCLA Medical Center; University of Georgia; Carnegie Mellon University; Benaroya Research Institute at Virginia Mason

## Abstract

Super-resolution optical imaging tools are crucial in microbiology to understand the complex structures and behavior of microorganisms such as bacteria, fungi, and viruses. However, the capabilities of these tools, particularly when it comes to imaging pathogens and infected tissues, remain limited. We developed μMagnify, a nanoscale multiplexed imaging method for pathogens and infected tissues that are derived from an expansion microscopy technique with a universal biomolecular anchor. We formulated an enzyme cocktail specifically designed for robust cell wall digestion and expansion of microbial cells without distortion while efficiently retaining biomolecules suitable for high-plex fluorescence imaging with nanoscale precision. Additionally, we developed an associated virtual reality tool to facilitate the visualization and navigation of complex three-dimensional images generated by this method in an immersive environment allowing collaborative exploration among researchers around the world. μMagnify is a valuable imaging platform for studying how microbes interact with their host systems and enables development of new diagnosis strategies against infectious diseases.

## Introduction

The small size of microbes, varying from 0.15 to 700 μm^1^, makes it challenging to investigate the spatial arrangements of biomolecules under conventional optical microscopy, which can only provide approximately 250 nm resolution. Electron microscopy offers atomic-level resolution but requires expensive instrument and lab setup, provides little molecular contrast, and involves tedious sample preparation including dehydration and chemical fixation as well as slicing thin sections of the tissue. Super-resolution imaging techniques evolved remarkably over the last decades to allow new microbiology observations, bringing the resolution down to 10–50 nm. However, these methods come with their own set of challenges such as long training times required for operating sophisticated systems or high costs related to purchasing and maintenance that present barriers, especially for laboratories with limited budgets^2^.

Expansion microscopy (ExM) is a recently emerged technique that offers remarkable resolving power without the need for expensive, specialized hardware. This approach involves embedding biological samples such as organs, tissues, and cells in superabsorbent polyacrylate gels and anchoring key biomolecules onto the polymer network. After mechanical homogenization of the sample-gel hybrids, they can be expanded isotropically by immersing them in an aqueous solution to decrowd the biomolecules. ExM has been continuously evolving with the improvements including large volume^3,4^, expansion factor^5^, and multiplexibility^6,7^.

Despite the impressive advancements in ExM, there has been limited research into microbial imaging applications thus far due to certain drawbacks of current methods: Lim et al. reported that mixed bacterial cultures show incomplete and heterogenous expansion patterns when treated with proteinase K and cell wall digestion^8^. This proteinase K treatment may also disrupt certain epitopes and limit multiplexing capability. Published expansion protocols for fungal and bacterial samples are all limited to 2–3 targets due to the need to construct fluorescent proteins fusion with their target^9^ or perform pre-expansion staining due to the use of proteinase K^10^. A recent study showed the possibility of combining heat denaturation with cell wall digestion to expand yeast cells^11^, potentially allowing for multiplexing. However, no methods have been demonstrated beyond infected cell cultures^10,12,13^, indicating a gap between single cell infection models and more complex scenarios, such as biofilms invading host tissues. Additionally few systematic characterizations of image distortion between pre- and post-expansion image pairs are available to other microbiology-focused ExM methods^14,15^, meaning that development of ExM for nanoscale microbiology imaging remains in its infancy stage overall.

We present μMagnify, a nanoscale imaging platform that enables high-plex fluorescence imaging of pathogen and infected samples. Derived from Magnify^16^, which universally anchors different kinds of biomolecules in the hydrogel, our optimized protocol combines heat denaturation with microbial cell wall digestion for homogenization. We validated our methods on different representative samples, such as bacterial and fungal supernatants, biofilm, infected cell cultures / tissue, with a less than 4% distortion and up to 8-fold expansion factor. Additionally, we demonstrated high-plex 3D nanoscale imaging of DNA, RNA, proteins, lipids, and polysaccharides in microbes through this method. Finally, an immersive visualization tool was designed allowing researchers around the world to perceive complex datasets from multiple angles, thus sharing knowledge in real time.

## Results

μMagnify expands a wide range of pathogens with minimal distortion.

Bacterial and fungal pathogens have rigid cell wall envelopes that play an essential role in maintaining cell shape and providing protection from osmotic pressure. The rigid structure of the microbial cell wall poses challenges for isotropic expansion using existing protocols. The common component of the bacterial envelope is a peptidoglycan layer consisting of long strands of glycans covalently crosslinked by stretchable peptides, while Gram-negative bacteria also have an additional outer membrane composed of phospholipids and glycolipids. Gram-positive bacteria lack this outer membrane but instead possess thicker layers of peptidoglycan for chemical resistance^17,18^. Fungal cells on the other hand feature polysaccharides including β−1,3 glucan, β−1,6 glucan, α−1,3 glucans or chitins with mannoproteins linked to them^19^.

**Figure 1:**
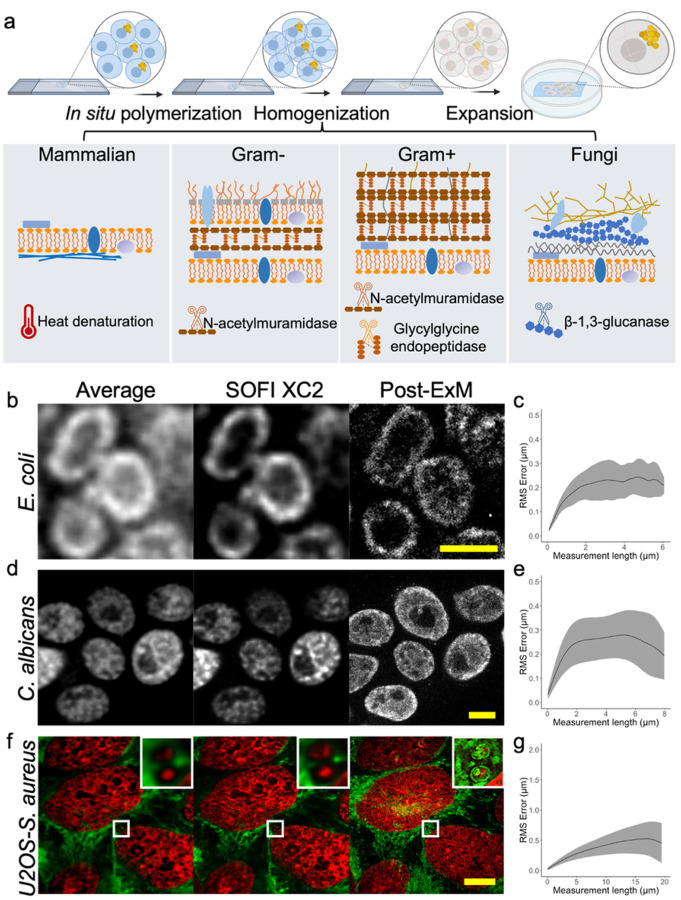
Schematic and validation of μMagnify. **(a)** Brief schematic of μMagnify chemical processing (Supplementary Figure 1). Briefly, samples were rehydrated and penetrated for format conversion. Then appropriate amount of gelling solution was added on top of the specimen for polymer synthesis. The sample-gel hybrid was mechanically homogenized in denaturing reagents followed by enzyme digestion. The homogenized gel was isotropically expanded in 1:50 diluted PBS or ddH2O for imaging. **(b-g)** Validations of μMagnify on representative bacteria, fungi, and bacteria infected cell culture pre- and post-expansion: (b) *E. coli* was stained with CellMask, (d) *C. albicans* with BODIPY and (f) *S. aureus*infected U2OS cell culture with DAPI (red) and BODIPY (green). **(b, d, f)**Examples of pre-expansion images taken at 60x and processed with intensity average across 50 frames and XC2 deconvolution SOFI, compared to the post-expansion images taken at 60x for the same field of view. Post expansion images are Maximum intensity projected over 5–30 frames in z to best match the plane. Biological scales: (b) and (d), 2 μm; (f), 10 μm. Expansion factor (in 1:50 diluted PBS): (b) 4.97 ± 0.32 (n=13); (d) 6.06 ± 0.42 (n=11); (f) 5.79 ± 0.21(n=16). Right top corner images in (f) are zoomed in images of the white boxed region (length = 2.6 μm). **(c, e, g)** Root mean square (RMS) length measurement error as a function of measurement length for pre-expansion SOFI images versus post-expansion images for (c) *E. coli* (CellMask, n = 13), (e) *C. albicans*(BODIPY, n = 11) and (g) *S. aureus* infected U2OS cell (DAPI, n = 16). Solid line, mean of channel; shaded area, standard error of mean (s.e.m).

Our goal is to develop an Expansion Microscopy approach optimized for studying biofilm and pathogen-host interactions in infected specimens. To achieve this, the method should possess certain capabilities: (1) expansion of a diverse range of specimens, including bacteria, fungi, and infected tissues; (2) preservation of diverse biomolecules allowing post-expansion staining; (3) a straightforward expansion process with resolution similar to traditional super-resolution imaging methods. We started by employing Magnify - a new Expansion Microscopy method that uses universal anchoring strategies to retain DNA, RNA, proteins, glycolipids and polysaccharides after expansion^16^. However, it soon became clear that its enzyme-free homogenization technique was not sufficient for expanding microbial cells. We also found that using reported enzyme-based homogenization^8–10, 12,13^ can only expand a small set of specimens, such as *E. coli*, at the cost of losing epitopes post-expansion. To address the issue of cell wall homogenization, we developed a cocktail of digestive enzymes specifically tailored to various types of cell walls (Fig. 1a). Mutanolysin (N-acetylmuramidase) was used to break down the 1,4-beta-linkages between N-acetylmuramic acid and N-acetyl-D-glucosamine residues in peptidoglycan for both Gram-positive and Gram-negative bacteria; lysostaphin (glycylglycine endopeptidase) was added to cleave the crosslinking pentaglycine bridges presented in Gram-positive bacteria; finally, zymolyase (β−1,3-glucan laminaripentao-hydrolase and β−1,3-glucanase activity) was included for digestion of fungal cell walls. To preserve epitopes for post expansion staining, no proteases such as proteinase K were included in this enzyme mixture.

We observed that incubation in denaturant-rich solution at 80°C followed by digestion with the enzyme cocktail successfully expanded a variety of bacterial and fungal specimens, including biofilms and infected cell cultures/tissues. We found no substantial morphological difference between pre- and post-expansion images of both bacteria and fungi (Fig. 1b,d). We named the new method, microMagnify (μMagnify), where micro-refers to microbial cells. We further confirmed a low distortion level (< 4%) achieved by μMagnify on all the specimens we tested, including Gram-negative and Gram-positive as well as bacterial and fungal samples, comparing between super-resolution optical fluctuation imaging (SOFI) pre-expansion^20^ and confocal microscopy post-expansion images (Fig. 1c, e, f & g, **Supplementary Fig. 2**). Particularly noteworthy is that our process increased lipid dye accessibility to previously intracellular Gram-positive bacteria which could not be stained before expansion without compromising the low distortion rate (Figs. 1f & g).

### μMagnify reveals intracellular structure of microbial cells with molecular contrast

**Figure 2:**
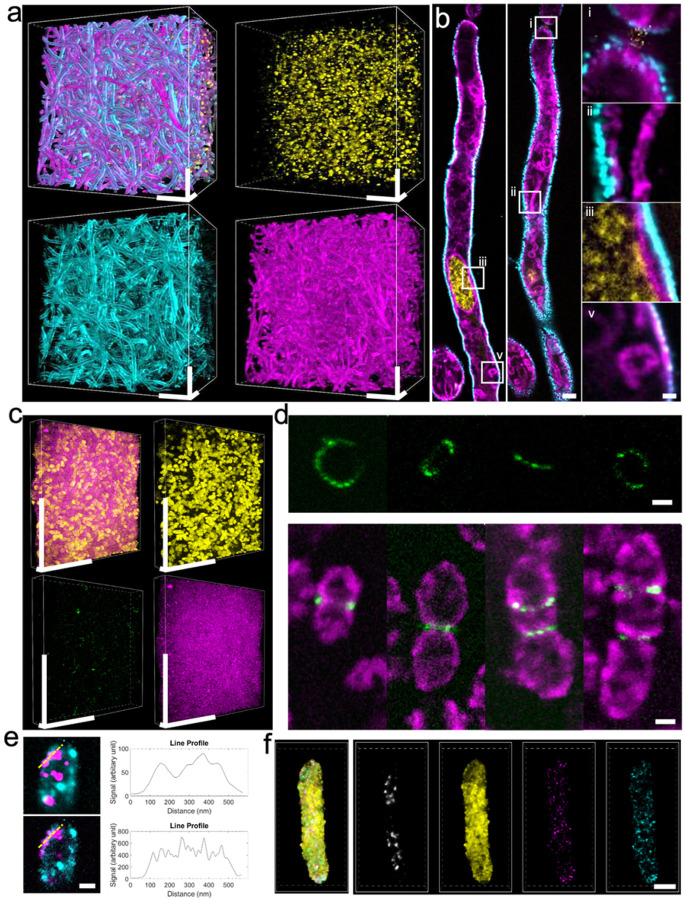
μMagnify works for a diversity of microbial cells, revealing their nanoscale structure and spatial patterns. **(a)** 3D reconstruction of fully expanded *Candida albicans* (*C. albicans*) biofilm. The sample was stained with DAPI (yellow), *Lycopersicon Esculentum* Lectin (LEL, cyan), and DiI (magenta). Physical scale: 100 μm in x, y, z. **(b)** X-y section of hyphae. Zoom-in views from boxed region showing the hyphae cell junction (i), elongated mitochondria beside cell wall (ii), details of nuclear membrane, cell membrane and cell wall (iii), and lipid body (v). Physical scales: 5 μm (most left and middle columns), 1 μm (most right column). **(c)** 3D reconstruction of *Streptococcus pneumoniae* (*S. pneumoniae*) biofilm. Sample was stained with DAPI (yellow), LEL (green) and NHS-ester (magenta). Physical scales: 100 μm (x, y), 10 μm (z). **(d)** Upper part: examples of ring-like structures of peptidoglycan (PG) enrichment of dividing *S. pneumonia* at different orientations. PG was stained with LEL. Lower part: examples of *S. pneumonia* constriction by PG at different dividing stages. Cells were stained with LEL (green) and NHS-ester (magenta). Physical scale: 2 μm. **(e)** Viral particles imaging in E. coli. The sample was stained with Wheat Germ Agglutinin (WGA, cyan) and JCV capsid VP1 antibodies (magenta). Upper part shows spatial distribution of the virus particles in the fully expanded *E. coli* and its intensity profile (upper right) along the yellow dashed line. Lower part shows SRRF processed image from the same ROI and its intensity profile (lower right) along the dashed line. Physical scale: 2 μm. **(f)** Simultaneous protein and RNA imaging of fully expanded *E. coli*, revealing the spatial distribution of mNeon proteins (yellow) and its mRNAs (magenta). The bacteria are also stained with DAPI (gray) and 16s rRNA for control (cyan). Physical scale: 5 μm. All the expansion factors were characterized in *Supplementary Table 1*.

μMagnify achieved up to 8-fold expansion factor (**Supplementary Fig. 3**, **Supplementary Table 1**), enabling nanoscale imaging of microbial cell morphology and intracellular organelles with traditional optical microscopes. By clearing opaque specimens and separating densely packed structures, μMagnify provides an effective approach to visualize dense bacterial or fungal biofilms, which have been challenging to image due to the presence of light-scattering extracellular matrix, light-absorbing biomolecules, and difficulties identifying individual cells/microcolonies within densely packed structures. This is demonstrated by successfully imaging individual cells in a ~ 500 μm thick *Candida albicans* (*C. albicans*) biofilm after expansion (Fig. 2a). Dil was also used as a lipophilic dye to stain the membranes of individual cells in the biofilm (Fig. 2b), highlighting fine structures such as the nucleus envelope and mitochondria. Furthermore, μMagnify successfully imaged densely packed *Streptococcus pneumoniae* (*S. pneumoniae*) biofilm (Fig. 2c) along with peptidoglycan (PG) stained by *Lycopersicon esculentum* Lectin (LEL) that revealed PG arrangements at septum rings in individual cells (biological size less than 1μm) at different dividing stages (Fig. 2d). The image resolution of μMagnify can be further improved by combining it with other computational super-resolution techniques without upgrading the microscope; for instance, when combined with SRRF image processing^21^, we were able to resolve capsid particles of human polyomavirus viral protein (VP1) overexpressed and self-assembled inside *E. coli* (Fig. 2e) measuring particle sizes around 40 nm, consistent with literature findings^22^.

μMagnify offers the unique ability to simultaneously image and study multiple types of biomolecules in microbial cells. This technique allows for a diverse range of samples to be studied, including proteins, nucleic acids, lipids, carbohydrates and more. As demonstrated by examples with *C. albicans* and *S. pneumonia* biofilms (Figs. 2a–b), where DNA, carbohydrate, proteins and lipids were imaged together; *E. coli* (Fig. 2f, **Supplementary Fig. 4**) which was labeled with mNeon fluorescent protein and its mRNA, and 16s rRNA after expansion; as well as JCV virus expressed in *E. coli* (Fig. 2e), which was labeled with anti-VP1 antibodies after expansion - μMagnify is capable of multi-modal imaging for comprehensive understanding of a sample’s composition. Furthermore, this technology provides researchers an unprecedented versatility that can help answer questions about different types of samples not achievable through traditional methods - thus expanding the scope of potential discoveries in microbiology research.

### μMagnify expands archival clinical tissue specimens with infection

Formalin fixation is commonly used to generate archival tissue specimens for clinical examination and pathology research. However, it is challenging for traditional ExM methods such as pro-ExM^23^ and MAP^3^ to expand these types of specimens due to heavy formaldehyde-induced peptidyl crosslinks. Previous work has demonstrated expansion of pathogen-free formalin-fixed specimens using aggressive proteinase K digestion but at the cost of losing protein epitopes. Derived from Magnify, μMagnify can expand formalin-fixed tissue specimens while preserving its protein epitopes after expansion. To maximize preservation, we optimized our protocol by exploring various conditions for homogenization and preincubation (**Supplementary Table 1**, **Supplementary Fig. 5a-d**). We found that heat denaturation before cell wall digestion was necessary to prevent the formation of cracks in the sample; neither doing heat denaturation after cell wall digestion (**Supplementary Fig. 5e-f**) nor performing proteinase K digestion (**Supplementary Fig. 5i-j**) provided better preservation than heat denaturation preceding cell wall digestion (**Supplementary Fig. 5g & h**).

**Figure 3:**
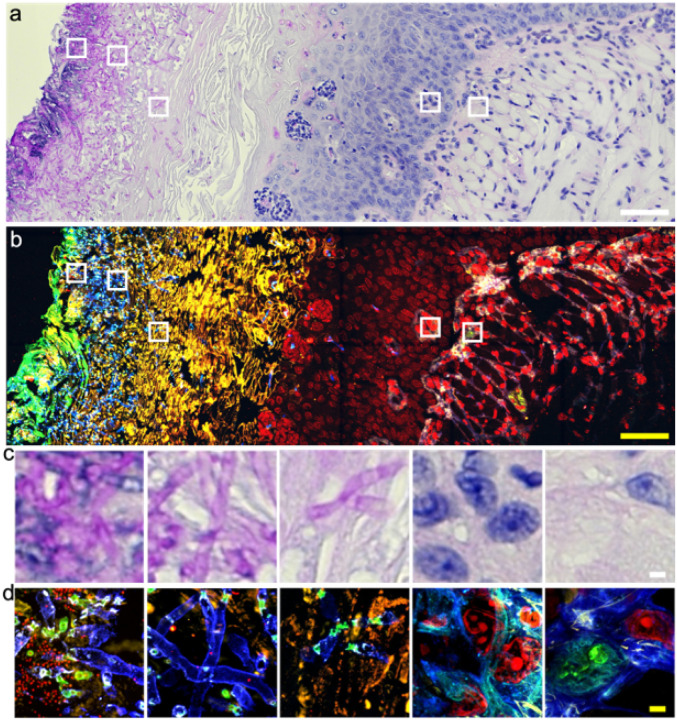
Expansion microscopy imaging of Candida albicans-infected FFPE mouse tongue tissue. **(a)**Bright field pre-expansion image of *Candida albicans* (*C. albicans*)-infected tissue that was stained with Periodic acid-Schiff stain (PAS). The cell wall of *C. albicans* were shown in magenta. Nucleus of the tissue is stained in blue. Scale bar: 50 μm. **(b)** Confocal fluorescence image of sample (a) expanded in PBS. Sample was stained with DAPI (red), cellular sugar molecules were labeled differently by WGA (cyan), and LEL (blue). Pan-proteins were labeled with NHS-ester (yellow). Biological scale: 50 μm. **(c)** Zoom-in views of the boxed regions in (a) from left to the right, showing gradient density of C. albicans infection. Scale bar: 2 μm. **(d)** Zoom-in views of the boxed regions in (b) from left to the right. Biological scale: 2 μm. Expansion factors were characterized in *Supplementary Table 1*.

We first tested μMagnify on formalin-fixed and periodic acid-Schiff (PAS) stained mouse tongue infected by *Candida albicans*. Bright field microscopic images revealed the presence of *Candida albicans* infection, highlighted by magenta staining from the polysaccharide-enriched cell wall, but lack of clear structural details in high magnification (Fig. 3a, c). We used μMagnify to expand the same slide and successfully stained DNA, proteins, and polysaccharides post-expansion (Fig. 3b, d), revealing detailed molecular structures that were not visible in the original PAS stain.

**Figure 4:**
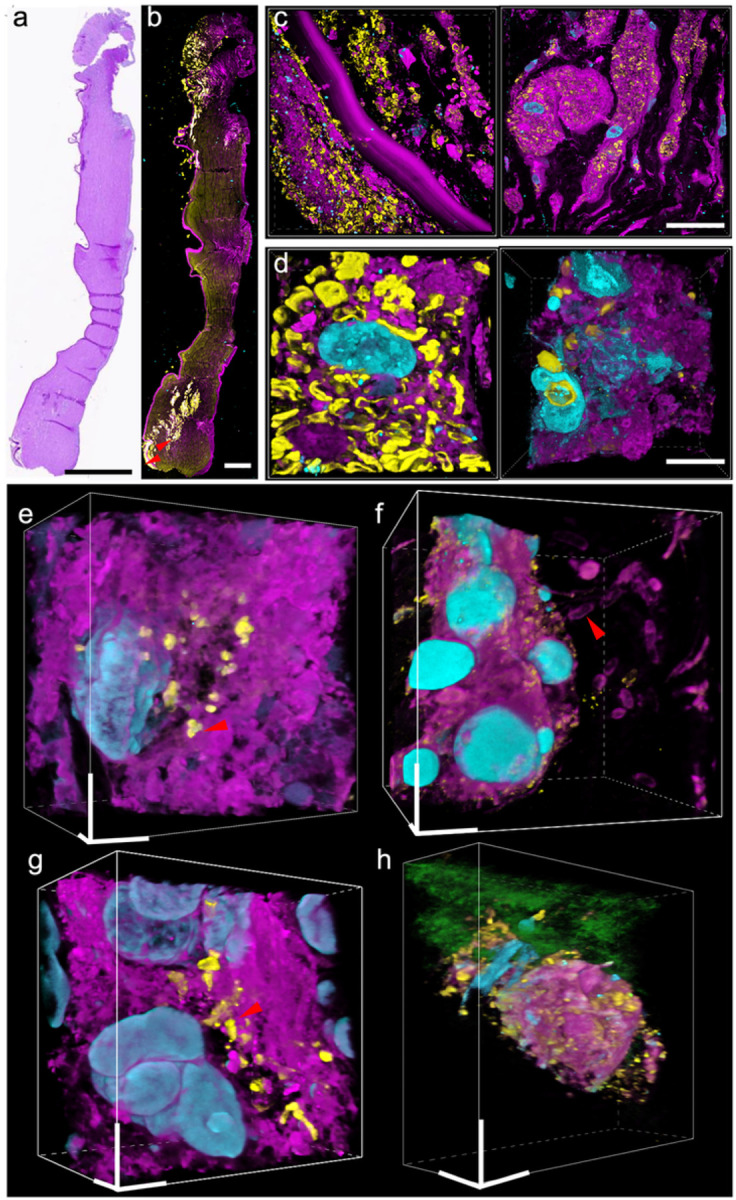
Nanoscale 3D characterization of various pathogen-infected cornea samples. **(a)** PAS image of FFPE cornea sample of candida keratitis. Scale: 1000 μm. **(b)** μMagnifyimage of tissue sample cut adjacently to that from (a), taken by 4x objective. Sample were post-expansion stained with DAPI (cyan), LEL (yellow), and NHS-ester (magenta). Scale: 1000 μm. **(c)** μMagnify images of pointed region in (b), taken at 10x objective, showing apparent distinction of local *Candida* infection (yellow). Scale: 25 μm. **(d)** Representative images of *C. albicans* interactions with normal (left) and immune (right) cells residing in cornea stroma. **(e-h)** Single-cell level characterizations for various types of eye infections. Samples were stained with DAPI (cyan), WGA (yellow), NHS (magenta), and LEL (green, in h). Scales: 10 μm (x, y, z). **(e)** example image of pathogen-host interactions in *Staphylococcus epidermidis*(*S. epidermidis*, Gram-positive) keratitis eyeball sample. **(f)**example image of extracellular pathogen in *Pseudomonas aeruginosa*(*P. aeruginosa*, Gram-negative) keratitis cornea sample. **(g)**example image of pathogen-host interaction in atypical mycobacterial (neither Gram-positive nor Gram-negative) keratitis cornea sample. **(h)** representative images of acanthamoeba located in cornea stroma. Expansion factors were characterized in *Supplementary Table 1*.

Keratitis is a sight-threatening disease, primarily caused by infectious agents such as bacteria, viruses, fungi and protozoa^24^. Diagnosing keratitis using histological techniques can be difficult due to the low contrast between pathological features and background tissue, poor resolution of images taken and complexity in identifying different forms of keratitis present within samples examined (**Supplementary Fig. 6a-e**). Traditional ExM methods with either proteinase K digestion or heat denaturation step do not work for human cornea tissues that consist primarily of dense collagen fibrils embedded in a proteoglycan-rich matrix; We modified the μMagnify protocol by supplementing collagenase into digestion buffer which proved capable of expanding infected human cornea sections up to 3.6× in PBS or 8× in water (**Supplementary Table 1**). Utilizing μMagnify enabled us to reconstruct 3D images of endothelium cells, epithelium cells and stromal keratocytes residing within the cornea structure itself (**Supplementary Fig. 6f-h**). Moreover, lectin stains combined with μMagnify enable distinction of different pathogens. For example, LEL strongly stains *Candida albicans* cell wall (Fig. 4a–d), comparable to H&E stain (**Supplementary Fig. 6a**); Wheat germ agglutinin (WGA) stain combined with morphological analysis can distinguish Gram-positive *staphylococcus epidermidis* cells (Fig. 4e, WGA^+^, round shape), which can be vaguely identified by H&E stain (**Supplementary Fig. 6b**), Gram-negative *pseudomonas* (Fig. 4f, WGA^−^, rod shape) that is hard to distinguish the early stage invasion (**Supplementary Fig. 6c**), *mycobacteria* (Fig. 4g, WGA^+^, rod shape), comparable to acid fast stain image (**Supplementary Fig. 6d**), and protozoan infections (Fig. 4h, WGA^−^ with much larger size than bacteria), comparable to H&E stain (**Supplementary Fig. 6e**). Without the need to use different dyes, μMagnify allows us to accurately differentiate between different forms of keratitis tissues within samples examined with a much higher resolution, thus paving way for potential new diagnostic methods for keratitis.

### Signal flunmixing enabled multiplexed imaging using μMagnify

μMagnify’s unique ability to retain biomolecules in a hydrogel allows for multiplexed super-resolution protein imaging in 3D tissues, circumventing the limitation of conventional techniques that struggle to detect more than four targets due to their broad excitation and emission bandwidth. Serial imaging is enabled by this method, allowing different sets of molecules from the same field of view to be imaged sequentially, enabling simultaneous characterization of various players during pathogen-host interactions. We used computational signal flunmixing algorithm^25^ to deconvolve overlapping signals from different rounds, instead of eliminating existing signals using photoinactivation^26^ or customized barcoded antibodies^27^. We accumulated the signals in raw images, such that true signal of current round is determined by the accumulated raw image subtracted signal from the previous round multiplied by a coefficient (**Supplementary Fig. 7a**). Enumeration within a range of possible coefficients will then reveal an optimal coefficient which minimizes the mutual information between the previous round image and true signal (**Supplementary Fig. 7b**).

We decided to use the multiplexing and resolution capabilities of μMagnify to visualize intracellular traffic of host proteins and microbes during bacterial infection. We mimicked *S. aureus* infection by treating a human cell osteosarcoma cell line (U2OS) with heat-killed *S. aureus* in combination with active *S. aureus* alpha-toxin. We stained the sample with nucleus (DAPI), two distinct groups of carbohydrates (WGA and ConA), lipid membrane (DiI), markers of the late endosome/MVB (CD63) and cytoskeleton (Vimentin, α-tubulin), and pan-protein (NHS ester conjugated Atto647N). To assess the ability to resolve protein relocalization and colocalization, cells were also stained with two proteins known to interact at endolysosomal membranes, the ubiquitin ligase NEDD4, and LITAF/SIMPLE^28^ (expressed as a GFP-tagged fusion and visualized with anti-GFP antibody).

**Figure 5:**
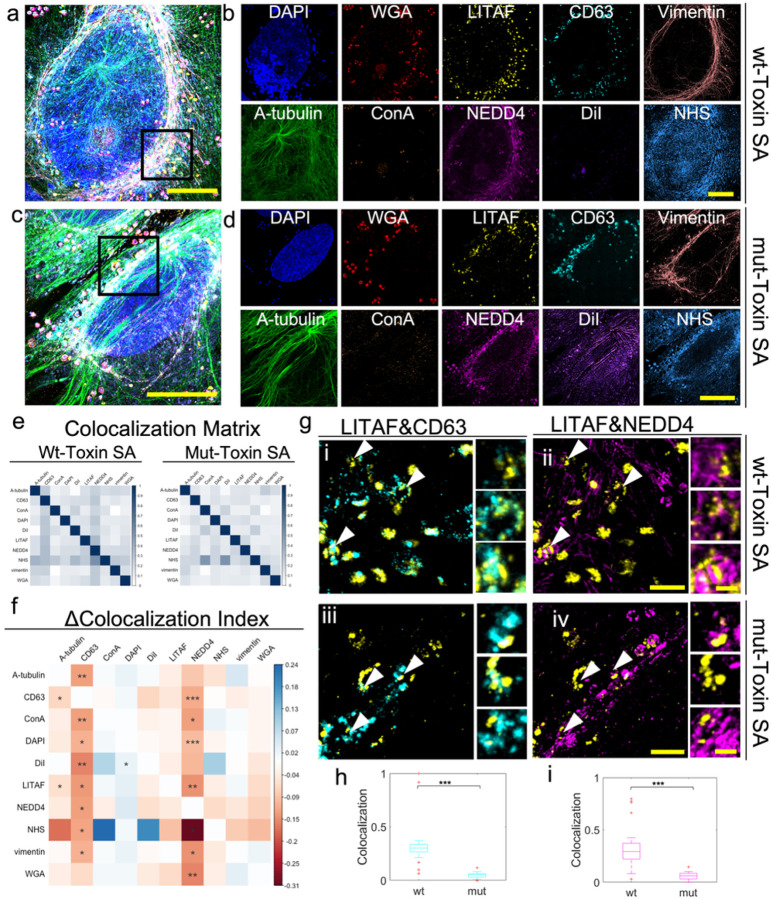
Multiplexing on *S. aureus*-infected U2OS cell culture allows pairwise study on protein-protein interactions. **(a)** Ten-color multiplexed imaging of α-toxin treated *S. aureus* infected wildtype U2OS cell. Sample was stained with DAPI, WGA, anti-GFP (targeting LITAF fusion with GFP), anti-CD63, anti-Vimentin, anti-α-tubulin, ConA, antiNEDD4, DiI, and NHS. Biological scale: 10 μm. **(b)** Single color images at different detection channels in (a). Biological scale :10 μm. **(c)** Ten-color multiplexed imaging of α-toxin treated *S. aureus* infected LITAF mutant U2OS cell. Sample was stained the same as those in (a). Biological scale: 10 μm. **(d)** Single color images at different detection channels in (c). Biological scale: 10 μm. **(e)** Colocalization matrices calculated for α-toxin treated S. aureus infected wildtype U2OS (left, n=3) and α-toxin treated S. aureus infected LITAF mutant U2OS cell samples (right, n=3) among 10 channels. For characterizing colocalization between signal1 and signal2. Colocalization coefficients are calculated as the percentage of the overlapping volume between the signal 1&2 in the volume of signal 1 or signal2. **(f)** Matrix of delta colocalization coefficient between Mut and Wt matrices in (e), indicating the change of pairwise signal colocalization among 10 channels. Asterisks indicated the significant levels through one-way ANOVA test, * p <0.05, **p<0.01, ***p<0.001. **(g)** Representative images of pair analysis for LITAF&CD63 and LITAF&NEDD4 colocalization. The first row showing images (i-ii) from boxed region in (a), the second row showing images (iii-iv) from boxed region in (c). The first column (i, iii) showing composite images of LITAF (yellow) and CD63(cyan). The second column (ii, iv) showing composite images of LITAF (yellow) and NEDD4 (magenta). Biological scales: 2 μm. Zoom-in views of arrow pointed regions (top to bottom) are listed on the right side of each image, delineating the different levels of colocalization between two signals. Biological scales: 500 nm. **(h-i)** Box plot of average colocalization coefficient between Wt (n=22) and Mut (n=16) for LITAF&CD63 (h) and LITAF&NEDD4 (i) in *S. aureus*-containing vacuoles. Middle line in the box shows the median. Bottom and top of each box show the 25^th^ and 75^th^ percentile of the data. Upper and bottom whiskers show the non-outlier maximum and minimum. Outliers are shown in red cross. Asterisks indicated the significant differences between Wt and Mut through one-way ANOVA test, ***P <0.001. Expansion factors were characterized in *Supplementary Table 1*.

All ten stains could be visualized in U2OS cells (Fig. 5a–d), and *S. aureus* could be seen at the cell surface or inside endosomes (Fig. 5a–d and **Supplementary Fig. 8**, **Supplementary Video 1**). The resolution of the image allows for reliable colocalization measurements of every marker simultaneously to investigate the change of protein-protein colocalization between different samples (Figs. 5e–f, **Supplementary Fig. 9**). To assess the accuracy of co-localization measurements, we measured the co-localization of LITAF with CD63 and NEDD4. It has been previously reported that LITAF localizes to CD63-positive late endosomes and multivesicular bodies (MVBs) through a mechanism that requires interaction with NEDD4^29^. Consistent with these reports, LITAF could be seen colocalized with CD63 and with NEDD4 in endosomal structures surrounding internalized *S. aureus* particles (Fig. 5g). Notably, co-localization was absent for a LITAF mutant that lacks the NEDD4 interaction PPxY motifs (LITAF Y23AY61A) (Fig. 5g, h–i).

### Immersive visualization of multiplexing data

**Figure 6:**
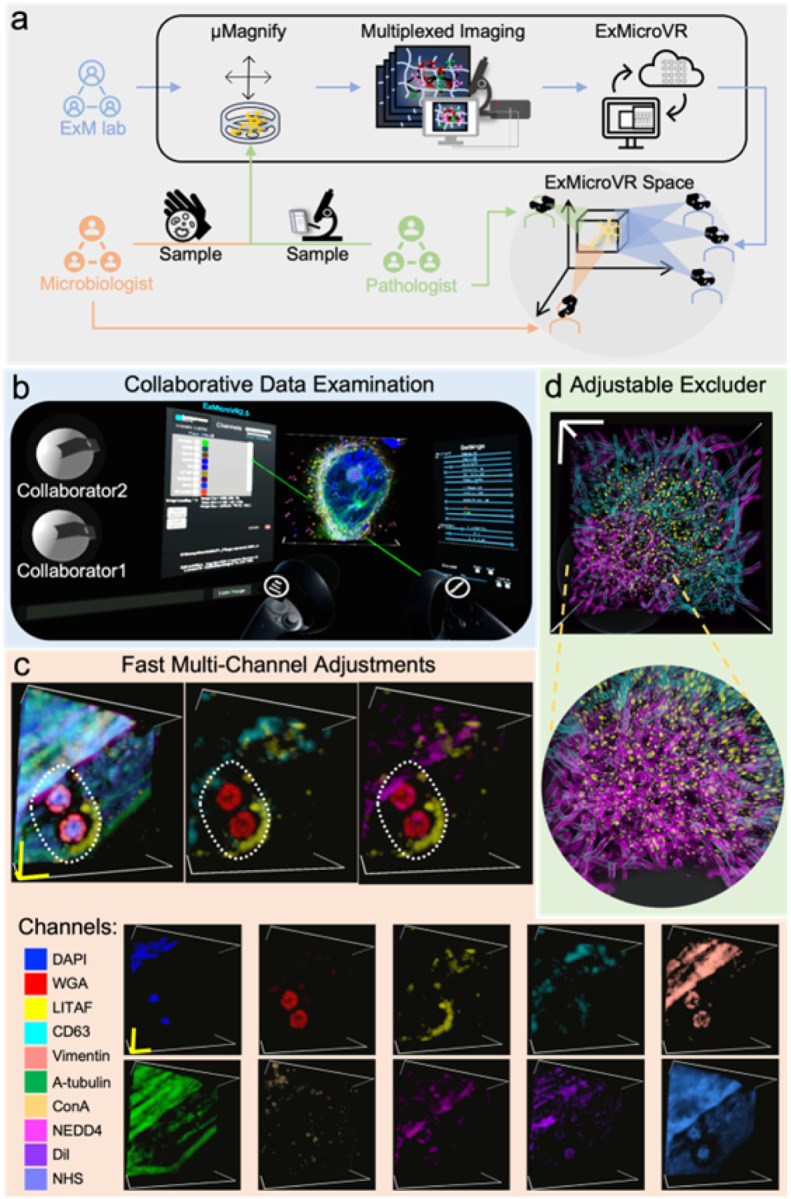
Immersive visualization of multiplexing data and collaboration through ExMicroVR software. **(a)** Workflow of collaboration among ExM, microbiology, and pathology research groups. Microbiologist and pathologist provide samples of infections along with list of potential biomarkers. ExM lab uses μMagnify to expand the samples and acquire multiplexed images. Images are converted to ExMicroVR-compatible format followed by data examination and interpretation through immersive visualization and real-time discussion in ExMicroVR space. **(b)** A representative user view of collaborative data examination through ExMicroVR. 3D multi-color Image data are presented and adjusted real-time among joined users. **(c)** Fast multi-channel data adjustments through ExMicroVR. Example images from mutant cell (figure 5c) that was stained with DAPI, WGA, antiGFP (targeting LITAF fusion with GFP), antiCD63, antiVimentin, antiAtubulin, ConA, antiNEDD4, DiI, and NHS. Biological scales 1 μm in x, y, z. Each channel is easily adjusted and color coded. Composite images can be made to study the interactions between different channels. **(d)** Size-adjustable excluder applies to inspection of thick biofilm data (figure 2a), Physical scale: 100 μm.

As μMagnify enables the collection of nano-scale multi-channel image-stacks for microbiology and pathology samples, we further developed a virtual reality (VR) application based on ConfocalVR^30^, called ‘ExMicroVR’ to provide the researchers immersive environment of data visualization and exploration that are inaccessible with previous software (Fig. 6a). Investigating micro-scale host-pathogen interactions is inherently a challenge of interpreting the 3D arrangement of nanoscale protein structures. In a VR environment, selective attention abilities were enhanced from both a behavioral and neural perspective^31^, suggesting that ExMicroVR may be a superior visualization approach comparing to those that typically used in research (e.g. desktop/laptop computers with 2D screens). A representative user view (Fig. 6b) as well as the video (**Supplementary Video 1**) demonstrates the ExMicroVR in operation. (Note: 2D videos do not capture the full immersive nature of VR renderings of these complex image-stacks.). Compared to ConfocalVR, we enhanced the functionalities of to accommodate the need to interact with the image stacks, control visualization, analyze the data, and share scientific insights in real-time among collaborators. The following new features were added: (1) Optimizing GPU processing load management. ExMicroVR can manage a multi-channel image-stack with 10 channels of 10^8 voxels. The processing load is split between your computer’s CPU and GPU, where possible processing performed on the highly parallel GPU. Furthermore, a comfortable video refresh rate can be maintained through using channel focus mode, selective excluders, and adjustable render quality; (2) Easy multi-channel adjustments (Fig. 6c): selection of channels of interested could be done by a single click while individual or all channels can have their own parameters adjusted; settings can also be saved and reloaded quickly; (3) Adjustable excluder ROIs improve viewing inner biological structures packed densely together such as biofilms (Fig. 6d); this also allows users to attach an excluder to their virtual ‘head’ so that areas in front are always clear while they move around within their images (**Supplementary Video 2**).

We demonstrated ExMicroVR to collect and visualize nanoscale structures of host-pathogen interactions from data associated with Fig. 5 in a collaborative VR environment. With ExMicroVR, we show where the structure of vacuoles around pathogens changes between toxin treated SA-infected and wild-type U2OS cells (Fig. 6c; **Supplemental Fig. 8**). The data generated using our μMagnify platform can provide the basis for further exploration in ExMicroVR, which offers interactive tools to display and analyze the complex 3D architecture of nano-scale structures from many perspectives with enhanced immersive visualization capabilities. This system is likely to be useful for understanding how pathogens interact with their hosts at nanoscale resolution to gain insights into pathology and foster novel treatments or strategies against infectious diseases.

## Discussion

We herein described μMagnify, a versatile nanoscale imaging method that enables identification and localization of various biomolecules of pathogen-infected samples, including proteins, DNA, RNA, lipids, and polysaccharides. We also conducted a valid characterization of the distortion that was not documented in other microbial expansion methods^8–10,12^. Without the need for dedicated anchoring^5,6^ or custom linkers to preserve biomolecules^13,32^, it is easy to apply μMagnify in a wide variety of pathogen samples, including Gram-positive, Gram-negative bacteria, fungus, virus-infected cell cultures and tissues. μMagnify is the first expansion microscopy method demonstrated on pathogen infected FFPE samples, enabling potential clinical applications. In addition, taking advantage of the small size and fast diffusion rate of its monomers makes it possible to expand thick tissue^33^ and even whole organ^3^.

The size-adjustable nature of the hydrogel makes μMagnify a convenient tool for macroscale and microscale inspection of pathogen-infected samples on conventional imaging system. For microscale biomolecule localization, μMagnify enables approximately 8-fold physical expansion at each dimension of the sample to achieve twice resolution enhancement comparing to other microbial expansion methods^8–11,13^. If combined with super-resolution microscopy, such as Stimulated Emission Depletion Microscopy^34^, Structural Illumination Microscopy^35^, Single-molecule Localization Microscopy^36^, or post-imaging processing methods such as SOFI^20^ and SRRF^21^, it could provide even more enhancement of the resolution. For macroscale tissue-level exploration, imaging the hydrogel in its shrunken state with high ionic-concentration buffer allows a speedy construction of tissue atlases using immunostaining or label-free strategy^37^. The combined macroscale and microscale information generated by μMagnify, may reveal previously inaccessible spatial patterns that improve diagnosis of pathogen diseases^38^.

The multiplexed volumetric 3D data generated by μMagnify and presented by ExMicroVR could lead to systematic analysis with a higher resolution and depth in the field of microbiology and pathology. With retention of diverse biomolecules, μMagnify may be used in combination with multiplexed protein, DNA and RNA imaging methods, such as immune-SABER^27^, Ab-oligo cyCIF^39^, MERFISH^40^, seqFISH+^41^ enabling characterization of the compositions and interactions between pathogens and its host in infected cells and tissues with sub-diffraction limit spatial resolution.

In conclusion, μMagnify is a facile and powerful tool for unearthing pathogen-infected tissues with nanoscale resolution enhancement. This method could enable more precise diagnosis and novel insights into how infectious diseases progress.

## Methods

### Reagents and reagent preparation.

The following reagents were used in this study: Paraformaldehyde (PFA, P6148, Sigma Aldrich), Ethanol (111000200, FHARMCO), Xylene (214736, Sigma Aldrich), Sodium acrylate (SA, R624, AK Scientific; sc-236893B, Santa Cruz Biotechnology), N-dimethylacrylamide (DMAA, 274135, Sigma Aldrich), Acrylamide (AA, A8887, Sigma Aldrich), N,N’-Methylenebisacrylamide (BIS, M7279, Sigma Aldrich), Tetramethylethylenediamine (TEMED, T9281, Sigma Aldrich) 4-Hydroxy-2,2,6,6-tetramethylpiperidine 1-oxyl (4xHT, 176141, Sigma Aldrich), Sodium chloride (NaCl, S6191, Sigma Aldrich), Phosphate buffered saline 10x solution (BP399–1, Fischer Scientific), Ammonium persulfate (APS, A3678, Sigma Aldrich), Potassium Persulfate (KPS, 216224, Sigma Aldrich), Methacrolein (133035, Sigma Aldrich), Ethylenediaminetetraacetic acid (EDTA, 0.5M, BDH7830–1, VWR), TritonX-100 (T8787, Sigma Aldrich), Tris-BASE (BP152–1, Fischer Scientific), Proteinase K (ProK, EO0491, Fischer Scientific), Sodium dodecyl sulfate (SDS, L3771, Sigma Aldrich), Urea (U5378, Sigma Aldrich), Glycine (G8898, Sigma Aldrich), Mutanolysin (M9901, Sigma Aldrich), Lysotaphin (L7386, Sigma Aldrich), Zymolyase (E1005, Zymo Research), Ethylene carbonate (EC, E26258, Sigma Aldrich), Dextran sulfate (50%, S4031, Sigma Aldrich), 20X SSC buffer (RNase free, AM9763, Fischer Scientific), Tween20 (P1379, Sigma Aldrich), Deoxyribonucleic acid, single stranded from salmon testes (Salmon DNA, D7656, Sigma Aldrich).

SA stock solution was prepared with a final concentration of 50%. ddH2O was added in several times with continuing agitation to ensure complete dissolution (NOTE: ensure enough waiting time for dissolution before volume calibration). Monomer solution is composed of 4% v/v DMAA, 34% SA, 10% AA, 0.02% BIS, 1% NaCl in 1x PBS and stored at 4°C before use. Heat denaturation buffer is composed of 1% SDS, 0.75% Glycine, 8M Urea, 25 mM EDTA, 500mM Tris-BASE in 2× PBS, pH 8.5 at RT. RNA fluorescence *in situ* hybridization (FISH) buffer is composed of 20% v/v EC, 10% dextran sulfate, 0.1% v/v Tween20, 100 μg/mL Salmon DNA in 2x SSC. RNA FISH wash buffer 1 is composed of 10% v/v EC in 2x SSC. RNA FISH wash buffer 2 is composed of 0.1% v/v Tween20 in 2x SSC.

### Cell culture preparation.

#### E. coli suspension.

DH10B E. coli carrying a pBad-mNeonGreen plasmid (for RNA FISH in figure 2) were grown overnight shaking at 37°C in LB Broth with 100 mg/L ampicillin and, if induced, with 100 mg/L arabinose. DH10B E. coli carrying a pBad-VP1 plasmid (for capsid VP1 post-expansion staining in figure 2) were grown in 3 mL LB Broth with 100 mg/L ampicillin shaking at 37 °C. After 4 hours, arabinose was added to a final concentration of 30 mg/L and the cultures were grown for 18 hours at 25 °C. To collect the cells pellet, cultures are centrifuged at 4000 g for 5 min. They were then resuspended in 4% paraformaldehyde in PBS 6.8 pH and incubated at room temperature for 30 min. The cells were then washed twice by being pelleted at 1500 g for 5 min and resuspended with PBS.

#### Candida albicans biofilm.

Frozen strain SC5314 was maintained in 15% glycerol frozen stocks at −80°C. Strains were inoculated in liquid YPG at 30°C overnight. Overnight cultures were diluted to an OD600 of 0.2 in 2 ml RPMI media (R5158, Sigma Aldrich) in 6 well culture plates containing 1.5 cm × 1.5 cm sized medical-grade silicone squares. After 90 minutes, the biofilm squares were dipped in sterile PBS to wash unadhered cells and placed in a new 6 well culture plate containing 2 ml of RPMI media. Biofilm cultures (for thick biofilm expansion in figure 2) were incubated at 37°C with orbital shaking at 60 rpm. After 24 h, biofilms were fixed with ethanol/4% formaldehyde.

#### Streptococcus pneumonia biofilm.

*Streptococcus pneumonia* strain D39^42^ (for biofilm expansion and PG characterization in figure 2) was grown from frozen stocks by streaking TSA-II agar plates supplemented with +5% sheep blood. After inoculation into Columbia broth, cultures were incubated at 37°C and 5% CO2 until OD600 reached around 0.05. Cultures (3mL each) were then added into 6 well chambers that contained coverslips. Biofilm growth was promoted by incubating at 37°C and 5% CO2 for 24 hours. Then, the media was carefully aspirated, and dishes were washed twice with PBS. Subsequently, the biofilm samples were fixed with 4% PFA for 20min. The PFA solution was removed, and the samples were again washed twice with PBS before storage at 4°C until further processing.

#### Infected Homo sapiens bone osteosarcoma cells.

U2OS cells were purchased from ATCC (Manassas, VA, USA), and were used at passage below 5. Cells were maintained in McCoy’s 5A media, supplemented with 2mM GlutaMAX, 100 U/ml penicillin and 100 μg/ml streptomycin (Gibco), and 10% FBS (Seradigm/VWR). All cells were maintained at 37°C with 5% CO2. LITAF mutant (Y23AY61A) was generated using consecutive quick-change mutagenesis following vendor protocol on plasmid LITAF-MycDDK (origene) (cagccactgtctcttcagcggatggaggtgcggatg and gcatgaatcctccttcggcttatacccagccagcgc). GFP-LITAF constructs were generated by cloning LITAF sequences (WT and mutant), inside pEGFP-C2 using TOPO cloning. Before transfection, 2×10^5 cells were plated on 6 wells plates and left overnight. Cells were then transfected with 4.5 μl of Fugene 6 (Promega) and 1.5 μg of DNA following manufacturer instructions. The next day, the cells were split at 5×10^4 per well on glass-bottom wells with removable chambers (Grace Bio-Lab, Sigma Aldrich) and treated after 24h with α-toxin at 500 ng/ml and *S. aureus* particles (molecular probes) for 1h, before fixation in 4% PFA for 15 min.

### Formalin-fixed paraffin-embedded (FFPE) infected tissue preparation.

#### Candida albicans infected mouse-tongue sample.

BALB/c mouse model of oropharyngeal candidiasis (SC5314) was developed as described in the paper^43^. Paraffin-embedded thin sections of the tongue were obtained and stained with periodic acid-Schiff stain for histopathology analysis in comparison with expansion images in figure 3. Details of sample preparations are described in **Supplementary note 1**.

#### Pathogen-infected eye sample.

Following Wills Eye Hospital Institutional Review Board approval, 5 pathogen-infected formalin-fixed Paraffin-embedded corneal tissues (one case each of *Candida parapsilosis*, *Pseudomonas aeruginosa*, *Staphylococcus epidermidis*, *Mycobacterium chelonae*, and Acanthamoeba keratitis) were retrieved from pathology files. 4 μm thick sections were cut and stained with the following stains: Periodic acid-Schiff stain (PAS, for *Candida* keratitis), Hematoxylin-Eosin stain (H&E, for *Staphylococcus* keratitis), Brown Hopps Gram stain (Gram, for *Pseudomonas* keratitis), Ziehl Neelsen acid fast stain (AFB, for *Mycobacterial* keratitis), and hematoxylin-eosin stain (H&E, for *Acanthamoeba* keratitis). Detailed staining protocols for special microorganisms were provided in **Supplementary note 2** (PAS staining protocol is described below). Appropriate controls were run for each stain. Adjacent cut for each case were processed with μMagnify for pathogen detection in figure 4.

### Deparaffinization for FFPE tissue.

For formalin-fixed Paraffin-embedded (FFPE) pathogen-infected tissue, samples were sequentially placed in a series of solutions: 2× xylene, 2× 100% ethanol, 95% ethanol, 70% ethanol, 50% ethanol and (finally) doubly deionized water. All of these steps were performed at room temperature (RT), 3 min each.

### Periodic acid-Schiff staining for FFPE tissue.

Deparaffinize and hydrate tissue slide to water. Oxidize the tissue in 0.5% w/v periodic acid (375810, Sigma Aldrich) solution for 5 minutes. Rinse in distilled water. Place the slide in Schiff reagent (3952016, Sigma Aldrich) for 15 min, as sections become light pink during this step. Rinse slide in lukewarm tap water for 5 min, as sections immediately become dark pink. Counterstain in Mayer’s hematoxylin (MHS32, Sigma Aldrich) for 1 minute. Wash in tap water for 5 min. Dehydrate the tissue slide and mount the sample with mounting medium (23–245691, Fisher Scientific) and coverslips.

### Pre-expansion immunostaining of pathogen infected U2OS cells.

After fixation, cell culture was permeabilized for 10 minutes in 0.5% TritonX-100 in 1× PBS at RT followed by blocking with SuperBlock Blocking Buffer (37515, Fisher Scientific) in PBS for 10 minutes at RT. Samples were then incubated in staining buffer with CellMask green (C37608, Invitrogen, for *E. coli*-infected U2OS), and BODIPY^™^ FL DHPE (D3800, Fisher Scientific, for *S. aureus* and *C. albicans*-infected U2OS) and DAPI (62248, Fisher Scientific, for *C. albicans*-infected U2OS) together with 3 hours at RT. Samples were then washed at least 3 times with washing buffer for at least 10 minutes each at RT.

### *In situ* polymer synthesis.

Immediately prior to gelation, the chemicals 4HT, APS, TEMED, and methacrolein were added to the monomer solution with a final concentration of 0.2–0.25% (w/v) APS, 0–0.25% (v/v) TEMED, 0.001% 4HT (w/v), and 0.1–0.25% (v/v) methacrolein (Supplementary Table 2). The solution was vortexed, and the sample were incubated with the gelling solution for 10~40min at 4 °C to allow the monomer solution to diffuse into the cell while preventing premature gelation. A gelling chamber was then constructed, consisting of spacers cut from #1.5 cover glass and a glass slide, placed backside down, on top of the cell culture glass that removed from the plate. The samples were incubated overnight in a humidified container at 37 °C to complete gelation.

### Sample homogenization and expansion with μMagnify.

After gelation, samples were trimmed and incubated in denaturant-rich buffer (1% w/v SDS, 8M Urea, 25 mM EDTA, 2× PBS, pH 7.5 at RT). Incubation time depends on sample type: pathogen-infected cell culture was incubated for 3 hours; pathogen-infected mouse tongue tissues were incubated for 54 hours; pathogen-infected eye samples were incubated for 96 hours at 80 °C with shaking until the completion of homogenization (i.e., the gelled tissue remains flat without bending or twisting in the solution). Homogenized samples were washed with 1% decaethylene glycol monododecyl ether (C_12_E_10_)/1x PBS for at 60 °C twice, at least 15 min per wash, followed by two washes at 37 °C, at least 15 min per wash to ensure complete removal of SDS. Samples were further incubated in enzyme solution for cell wall digestion of the pathogens (Supplementary Table 1). To improve the expansion factor for thicker samples longer digestion times were required. Finally, gels were placed in ddH2O, 1:50 PBS or 1:50 SSC at RT for 10 min to expand. This step was repeated three to five times until the size of the expanded sample stabilized. Samples can be stored in 1× PBS containing 0.02% sodium azide at 4 °C.

### Post-expansion immunostaining.

Samples were incubated with primary antibodies (1:200 dilution) in 2× SSC (300 mM NaCl, 30 mM sodium citrate, pH 7.0)/1% Tween 20 for at least 3 hours at RT: rabbit polyclonal anti-α-tubulin (11224–1-AP, Proteintech), chicken polyclonal anti-vimentin (ab24525, Abcam), Chicken polyclonal anti-GFP (ab13970, Abcam), rabbit polyclonal anti-NEDD4 (PA5–17463, Invitrogen), mouse monoclonal anti-human polyoma virus JCV capsid protein VP1 (ab34756,Abcam), mouse monoclonal anti-CD63 (ab8219, Abcam). Samples were washed four times with PBS for 15 min each at RT, followed by secondary antibodies (1:500 dilution) incubation together with other fluorescent dyes (1:500–1000 dilution) in PBS for 1–3 hours at RT: Donkey Anti-Mouse IgG (H+L) CF^®^647 (20177, Biotium), Goat anti-Chicken IgY (H+L) Alexa Fluor^™^ 488 (A11039, Invitrogen), F(ab’)2-Goat anti-Rabbit IgG (H+L) Alexa Fluor^™^ 546 (A11071, Invitrogen), DAPI (62248, Thermo Scientific), CellMask^™^ green (C37608, Invitrogen), BODIPY^™^ FL DHPE (D3800, Invitrogen), Vybrant ^™^ DiI (V22885, Invitrogen), Lycopersicon Esculentum (Tomato) Lectin DyLight^™^ 649 (LEL, DL-1178, Vector Labs), DyLight^™^ 488 (DL-1174, Vector Labs), Concanavalin A Alexa Fluor^™^ 488 (ConA, C11252 ,Invitrogen), Atto 647N NHS ester (18373, Sigma), Sulfo-Cy3 NHS ester (GC17345, GlpBio), Wheat germ agglutinin CF640R (WGA, 29026, Biotium), WGA Alexa Fluor^™^ 488 (W11261, Invitrogen). Note that over 3 hours incubation of secondaries will cause potential non-specific binding.

### μMagnify RNA FISH.

Each target gene requires from 20–30 probes that consists of gene binding and adaptor regions^44^.The gene binding region of RNA FISH probes were designed by LGC bioresearch technologies’ Stellaris^®^ RNA FISH Probe Designer (https://www.biosearchtech.com/support/tools/design-software/stellaris-probe-designer). Probe sets were synthesized from IDT on a 96-well PCR plates. Each gene has a unique fluorophore-modified imager probes for identification (Supplementary Table 3) The RNA FISH protocol^45^ was modified: the sample was incubated at washing buffer1 at RT for 30 minutes. Prepare probe mixtures by diluting in hybridization buffer1 at a total probe concentration of 100 nM per gene. Vortex to mix. Probe mixture was added in the gel for 2–3 hrs incubation at 37°C. Samples were rinsed in washing buffer1 at 37°C, followed with two washes with washing buffer2 at 37°C and RT. Samples were expanded in 0.02 SSC right before imaging.

### Confocal Imaging.

Fluorescence imaging was performed using a Nikon Eclipse Ti2 epifluorescence microscope equipped with a CSU-W1 spinning disk confocal module and an Andor 4.2 Zyla sCMOS camera. The system was controlled by NIS-Elements AR 5.21.03 64-bit software. Images were taken using the following Nikon objectives: CFI Plan Apo Lambda 4× (0.2 NA), CFI Plan Apo Lambda 10× (0.45 NA), CFI Apo LWD Lambda S 20×WI (0.95 NA), CFI Apo LWD Lambda S 40×WI (1.15 NA), CFI Plan Apo Lambda 60×Oil (1.4 NA). DAPI was excited with a 405 nm laser and imaged with a 450/50 emission filter, Alexa Flour 488 was excited with a 488 nm laser and imaged with a 525/40 emission filter. Alexa Fluor 546 was excited with a 561nm laser and imaged with a 607/36 emission filter. Alexa Fluor 647 was excited with a 640nm laser and imaged with a 685/40 emission filter. During imaging, the gels were placed in glass-bottom customed plates with all excess liquid removed. Poly-L-lysin coating is recommended recycled imaging plates.

### SOFI image preparation.

Before evaluating expansion distortion (figure1 b–g), super-resolution optical fluctuation imaging (SOFI) method is applied to improve the image resolution of the pre-expansion image. This method takes higher order statistical analysis of stochastic temporal fluorescence fluctuations of emitters recorded in a sequence of images. By taking the *n*th-order cumulant of the original pixel time series, the fluorescence signal of the emitters within the pixel is preserved due to higher correlation values across time series, leading to an increased resolution. SOFI images were taken with CFI Plan Apochromat VC 60×C WI (1.2 NA). Each SOFI image consisted of 50–100 frames per z-plane with 100 ms exposure time per frame. SOFI images were processed using custom MATLAB code. Images were corrected for drift and intensity, cropped, and deconvolved (Lucy-Richardson method) after 3-dimensional cross-correlation SOFI.

### Measurement of the expansion factor.

For samples that have pre- and post-expansion image pairs, expansion factors were determined by SIFT key points distance (details in next section). For *E. coli* suspensions, expansion factors were estimated by average particle areas. Bacterial particle areas were determined using the Analyze Particles tool in FIJI/ImageJ after image thresholding and binarization. To calculate the linear expansion factor, the square root of the ratio of the average post-expansion to average pre-expansion particle area was calculated. For *S. pneumonia* biofilm and *C. albicans* biofilm, expansion factors were estimated by average cell width. Cell widths were determined by manually measuring line length in Analyze FIJI/ImageJ. Ratio of average length pre- and post-expansion were calculated by randomly sampling the images, as estimated liner expansion factor. For FFPE eye tissue, the expansion factor was calculated by measuring the tissue size, pre- and post-expansion. Expansion factors for each type of sample were used to normalize post-expansion images into biological scale. Normalization process also nulls out the small (<10%) natural sample-to-sample variability of the expansion process, as the ‘biological’ length units are obtained out of the total pool of field of views of the same type of samples.

### Measurement error quantification.

Distortion vector filed was generated to calculate the root-mean-square (RMS) error as previously described^46^. Briefly, for the same fields of view, pre-expansion SOFI images were taken at a single z-plane at 60× magnification and post-expansion images were obtained with multiple z-planes at 60× magnification. To find the best matching post-expansion z-planes, scale invariant feature transform (SIFT) key points were generated for all possible combinations of pairs of the pre-expansion images and post-expansion z projections. Different expansion factors and imaging conditions might lead to one post-expansion z projection from 5–30 z planes that corresponds to the pre-expansion z. SIFT key points were generated using the VLFeat open-source library and filtered by random sample consensus (RANSAC) using a geometric model that only permits rotation, translation, and uniform scaling. The pair of pre-expansion and post-expansion images with the most SIFT key points were then used for image registration, uniform scaling, calculation of expansion factors and distortion vector fields. Manual rigid image alignment by FIJI ImageJ was applied, when the SIFT algorithm fail to recognize appropriate number of SIFT key points. By subtracting the resulting vectors at any two points, distance measurement errors could easily be sampled, and the RMS error for such measurements was plotted as a function of measurement length from at least three technical replicates.

### Multiplexed fluorescence imaging for U2OS cells.

Our gel-sample hybrid was subjected to multiple rounds of staining without stripping off the previous-round staining, for the maximum preservation of the targeting biomolecules. This approach also speeds up the staining process for multiplexing. Wildtype and mutant U2OS cells were gelled and homogenized, followed by three cycles of post-expansion immunostaining and confocal imaging. Gel was expanded in 1:50 PBS before each round of imaging. For accessing the same ROI at every round of imaging. A stitched map image was first captured at 4x magnification with multipoint mode. According to the map, ROIs for each individual cell was found and imaged at 60x magnification with DAPI as the reference channel for image registration.

### Multiplexed fluorescence image processing.

After acquisition of three rounds of images for a batch of ROIs, signals from the spectrum-overlapping antibodies in each channel accumulated after each round of staining and imaging, which requires signal flunmixing to specify signals that belong to each biological target. First, images for each ROI were aligned by SIFT rigid registration by rotation, translation, and uniform scaling as mentioned above. For minor local misalignment, images were then subjected to diffeomorphic demons registration^47^. The displacement filed was calculated between DAPI channels from the two consecutive rounds to transform images from other three channels. Registered images were then processed with signal flunmixing to parcel out signals from individual markers. We customized a signal flunmixing algorithm. The signals between the two consecutive rounds (ImageR1 and ImageR2) follows this relationship: Image R_i_ = α*Image_i-1_+’True’Ri (equation 1). If the flunmixing is done, the mutual information between ImageR1 and subtracted ImageR2 should be minimum, considering the stains are targeting two unique biological molecules (equation 2). Therefore, there exists an optimal α factor that makes the mutual information between ImageR1 and ImageR2- α*ImageR1 minimum, we could use this α_opt_ to subtract ImageR1 from ImageR2 to get the pure signal of the second staining (equation 3).


(equation 1) $${\text{Image}}_{\text{i}}={\text{ImageR}}_{\text{i}}+\alpha *{\text{Image}}_{\text{i-1}}$$

(equation 2) $$\alpha_{\text{opt}}=\underset{\alpha }{\text{argmin}I}\left({\text{Image}}_{\text{i-1}};{\text{Image}}_{\text{i}}-\alpha *{\text{Image}}_{\text{i-1}}\right)$$

(equation 3) $${\text{ImageR}}_{\text{i}}={\text{Image}}_{\text{i}}-\alpha_{\text{opt}}*{\text{Image}}_{\text{i-1}}$$

### Colocalization Analysis.

To investigate the interactions among ten channels within each cell, we conducted a colocalization analysis. After background subtraction, registered 10-color image stacks were converted into a 4D matrix with the fourth dimension representing different channels. The colocalization of each possible two-channel combinations out of the ten channels were quantified to generate a 10×10 colocalization matrix. First, each channel data, as a 3D matrix was binarized by adaptive local thresholding with adjusted sensitivity (according to the SNR of each channel). For each colocalization test, particle1 from matrix1, particle2 from matrix2 and colocalized particles between two matrices were defined, labeled, and filtered (according to the SNR of each channel) by 26-connectivity. Colocalization index were calculated as the volume percentage of overlapping particles in particle1 and particle2. Colocalization matrix was generated by averaging the colocalization index across different ROIs for wildtype and mutant sample. Then a delta-colocalization matrix was calculated to show the change of colocalization between each two channels that was caused by the mutation in the cell. Particularly, we also interested in the colocalization between LITAF and NEDD4/CD63 in SA-containing vacuoles. We use FIJI ImageJ to manually crop those vacuoles from wildtype and mutant cells and run the colocalization test for LITAF&NEDD4 and LITAF&CD63 channels.

## Data Availability

Shared repository for μMaginfy generated image-stacks can be found at https://www.benaroyaresearch.org/our-research/programs/systems-immunology-division/expansion-microscopy-vr/expansion-vr-downloads. Other data are available upon reasonable request to the corresponding author of the paper.
